# Treatment-limiting decisions in patients with severe traumatic brain injury in a Norwegian regional trauma center

**DOI:** 10.1186/s13049-017-0385-x

**Published:** 2017-04-26

**Authors:** Annette Robertsen, Reidun Førde, Nils Oddvar Skaga, Eirik Helseth

**Affiliations:** 1 Department of Anesthesiology and Critical Care, Oslo University Hospital, Ullevål, P.O.Box 4950, Nydalen, N-0424 Oslo, Norway; 2 Department of Clinical Medicine, University of Oslo, Oslo, Norway; 3 Centre for Medical Ethics, University of Oslo, P.O.Box 1130, Blindern, N-0318 Oslo, Norway; 4 Department of Neurosurgery, Oslo University Hospital, Ullevål, P.O.Box 4950, Nydalen, N-0424 Oslo, Norway; 5 Oslo University Hospital Trauma Registry, Oslo, Norway

**Keywords:** Withholding treatment, Futility, Potentially inappropriate treatment, Traumatic brain injury, Decision-making

## Abstract

**Background:**

Treatment-limiting decisions (TLD) for severe traumatic brain injury (sTBI) have been sparsely studied. This study determine prevalence, main reason for, categories and timing of TLDs in a Norwegian regional trauma setting.

**Methods:**

A retrospective study of a 2-year cohort of 579 sTBI patients admitted to Oslo University Hospital (OUH). Prospectively collected data in the OUH Trauma Registry were combined with retrospective data from a chart review regarding TLDs.

**Results:**

TLDs were documented for 101/579 sTBI patients (17%). The situation was evaluated as futile in 59 cases and as potentially inappropriate in 42 cases. The three most frequent types of TLDs were withholding of neurosurgery, do not resuscitate orders and withdrawing of organ support. In 70% of cases, the first TLD was made within 2 days after injury, while in 14%, the first TLD was made later than day 7. Twenty percent (20/101) of the first TLDs were later adjusted, revoked in 4 patients and broadening of TLDs in 16 patients. The median time from the decision to death was 2 days (range 1–652). TLDs were documented in 93% of in-hospital death cases (*n* = 79). In-hospital deaths occurred in 73% of TLD group cases and 1% of non-TLD group cases. Family interaction and multi-team discussions were documented in >88% of cases, but no advanced directives were found, and notifications of patients’ preferences were found in only 7% of cases.

**Discussion:**

Clinicians should consider limiting treatment if continued treatment is not in the patients best interest. A range of different types of TLDs were applied for patients after sTBI in the trauma hospital setting.

**Conclusion:**

TLDs were found in 17% of sTBI patients. Value considerations behind TLDs in this care context need to be further explored.

## Background

Modern trauma care, including neurosurgery and life-sustaining therapies in intensive care units, is essential for survival after severe traumatic brain injury (sTBI). In Norway, the law provides physicians with decision-making authority for patients who lacks capacity [1]. Deciding when to continue, limit or withdraw life-sustaining treatment is an integral part of attending physicians’ responsibility [2–4]. Prolonged treatment may be assessed as futile immediately after the initial medical assessments, or futility may be recognized days, weeks or months after injury. Sometimes, there is considerable uncertainty about the achievable outcome of advanced treatment. Concern has been raised about the risks of overly pessimistic physicians, premature or paternalistic decision making in intensive care units (ICUs) and other barriers for optimal end-of-life (EoL) care [5–8]. Repeated communication between the care team and relatives about possible and realistic goals is essential to avoid conflicts and optimize support for families [9]. According to recent recommendations from the European Society for Intensive Care Medicine and the Society of Critical Care Medicine, the concept futility should be used only when the physiological goal cannot be accomplished, while the concept of potentially inappropriate treatment should be applied in more value-laden cases [10]. Recommendations have been made about optimal decision making processes with regard to withholding or withdrawing treatment for brain-injured patients [11–13]. The Neurocritical Care Society suggests a delay for at least 72 h with full physiological support before withdrawal of treatment to allow sufficient opportunity for prognostic evaluation, care planning and considerations of organ donation [11]. Different strategies have been described for addressing prognostic uncertainty, improving communication and optimizing the timing of treatment-limiting decisions both in the ethics and critical care literature [14–19]. Decisions to limit treatment are common in intensive care units (ICUs), but research shows wide variations in practice [20–24]. Scandinavian data on the prevalence of treatment-limiting decisions in brain-injured patients have not previously been published.

The aim of this study was to determine the prevalence, main reasons for, categories and timing of treatment-limiting decisions (TLDs) for patients with sTBI in a Norwegian regional trauma center setting.

## Methods

Oslo University Hospital (OUH) serves as the regional trauma care facility for approximately 2.9 million people. Geographically, the catchment area for the trauma facility is 110,000 km^2^. Patients with severe trauma are usually transported directly to OUH, and they are always transported there if suspected to be in need of urgent neurosurgical care, whereas seemingly fewer severely injured patients are treated at other hospitals in the region and transported to OUH if needed after consultation.

The OUH Trauma Registry (TR-OUH) prospectively includes all patients with an Injury Severity Score (ISS) ≥10, whether they are admitted to OUH directly or via a local hospital within 24 h after injury. Moreover, the registry includes all patients admitted under the auspices of the trauma team, penetrating injuries toward the torso, and/or injuries proximal to the elbow or knee, irrespective of ISS. The trauma team is alarmed on admission of patients who are obviously severely injured, unstable (circulatory/respiratory instability or reduced level of consciousness), victims of high-energy trauma, or in other situations with a high index of concern. The two registrars are experienced nurse anesthetists who have trauma team experience and are formally educated in coding the Abbreviated Injury Scale (AIS), the ISS and the New Injury Severity Score (NISS) [25–27]. The registry continuously undergoes validation and quality control by two senior anesthesiologists in charge of the registry. The registry is used for auditing, continuous quality improvement and research [28].

The study included all adult patients (>17 years) who had a head AIS 98 severity score of 4, 5 or 6 (severe, critical or unsalvageable TBI), with or without non-cranial injuries, fulfilled the criteria for registration in the TR-OUH, and were admitted to OUH in the time period Jan 1^st^ 2011 to Dec 31^st^ 2012, comprising a cohort of 579 patients.

No exclusion criteria were used. All patients admitted were included, irrespective of whether they died in the emergency room (ER) or following admission to the ICU or ward. Patients with an AIS severity score >1 (range: 1 to 6) in at least one body region other than the head, were categorized as multiple trauma patients, in contrast with patients with isolated head injuries [28]. Brain death patients were not excluded as we expected presence of TLDs prior to verification of brain death. An objective verification of total cessation of cerebral blood flow using cerebral angiography is required in Norway for the diagnosis of brain death.

According to Norwegian guidelines, physicians are obliged to document the most important ethical aspects of decision-making processes if life-sustaining treatment is withheld or withdrawn [1]. A study database was developed in FileMaker Pro v. 14 combining trauma registry data and clinical data retrieved from medical records (FileMaker Inc., Santa Clara, CA 95054 USA). Treatment trajectories for individual patients and logged decisions about TLDs were retrospectively retrieved from medical records. To assess data validity, a definition guide for the study variables was developed.

### Main reason for TLDs

The main reason behind each decision was coded based on the authors’ interpretation. Patient treatment prior to any TLD was categorized as either futile or potentially inappropriate. In the brain trauma setting, futility may be related to critical bleeding, cardiac arrest, devastating clinical neurological presentation, devastating CT scan pathology, intracranial hypertension, brain death or a combination of these entities. Potentially inappropriate treatment may be related to the degree of brain damage, prognostic uncertainty, significant comorbidity, explicit patient preferences or a combination of these entities.

### Categories of TLDs

The TLDs were categorized as follows: 1. withholding surgery, 2. withholding access to ICU, 3. withholding organ support, 4. do not resuscitate (DNR) orders, 5. no escalation of treatment, 6. withdrawing intracranial pressure-targeted treatment, 7. withdrawing organ support and 8. withdrawing nutrition (after being weaned off the ventilator). For one patient, several TLDs could apply. The location of the patient at time of the decision was registered: 1. ER, 2. ICU or 3. Ward. The time of decision was related to the time of injury and the time of death. If a sequence of TLDs was made for a patient, only the time of the first decision was noted.

### Dichotomizing TLD cases into withhold or withdraw

Cases were dichotomized based on dominant decisions type (mutually excluding categories): 1. only withholding life-sustaining interventions, 2. withdrawing life-sustaining interventions (may include withholding). Labeling was done based on decisions confronting clinicians prior to death regardless of mode of death.

### Decision-making processes

Based on the chart review, documented key aspects of decision-making processes were registered, such as multi-disciplinary discussions, family meetings, prognostic statements, explorations of the patient’s values and preferences, shared or unilateral decision-making and rationale for decisions. Documentation indicated whether there were disagreements or conflict between the treatment team and the family and whether palliative care consults or clinical ethical committees (CEC) were involved.

### Mortality

In-hospital mortality, 30-day mortality (30 days after injury) and 2-year mortality were registered.

### Statistics

Microsoft Excel v. 2010 (Microsoft Corp., Redmond, WA 98052–6399, USA) and SPSS v. 23 (SPSS Inc., Chicago, USA) were used for the data analysis. Chi-square test or Fisher’s exact test were used for categorical variables and unpaired *t* test or Wilcoxon rank sum test for numerical data to calculate a p value with a statistical significance level of 0.05. Survival functions were estimated by the Kaplan-Meier method.

## Results

### Study cohort

The study cohort consisted of 579 patients with a head AIS 98 severity score 4–6. The mean age was 53 years (range 18 – 97), 73% were male, a fall was the most common mechanism of injury (59%), 48% were multiple trauma patients, 85% were admitted to the ICU, and 49% required mechanical ventilation. Further patient characteristics are presented in Table 1.

**Table 1:** Cohort characteristics (*N* = 579). Comparison of patients with and without TLDs

Cohort characteristics		TLD-group (*N* = 101)	No TLD-group (*N* = 478)	*P* value
Sex and age	Male	75 (74%)	350 (73%)	0.831
	Female	26 (26%)	128 (27%)	
	Mean age (range)	60.8 (18–97)	51.3 (18–95)	<0.001
Mechanism of injury	Transport	23 (23%)	122 (26%)	0.562
	Fall	65 (64%)	274 (57%)	0.192
	Violence	3 (3%)	50 (10.5%)	0.018
	Self-inflicted	8 (8%)	8 (2%)	0.001
	Sport	3 (3%)	27 (6%)	0.270
	Other	1 (1%)	11 (2%)	0.401
GCS*	GCS 3 -8	70 (69%)	110 (23%)	<0.001
	GCS >8	31 (31%)	368 (77%)	
Injury severity	AIS* head max mean	4.8	4.4	<0.001
	ISS* median (range)	30 (17–75)	26 (16–75)	<0.001
	NISS* median (range)	57 (18–75)	38 (16–75)	<0.001
Intensive care	ICU* admission	86 (85%)	409 (86%)	0.914
	Ventilator days median (range)	6.3 (1–51)	11 (1–45)	<0.001
Neurosurgery	ICP* monitor	31 (31%)	159 (33%)	0.617
	EVD*	17 (17%)	21 (4%)	<0.001
	Craniotomy	18 (18%)	113 (25%)	0.240
	Hemicraniectomy	9 (9%)	17 (4%)	0.018
Hospital LOS*	Median (range)	3 (1–51)	6 (1–56)	0.04
Discharge destination	Home	0 (0%)	128 (26.8%)	<0.001
	Rehabilitation	2 (2%)	88 (18.4%)	
	Ward other hospital	7 (7%)	148 (31%)	
	ICU other hospital	12 (12%)	90 (18.8%)	
	Nursing home	6 (6%)	19 (4%)	
	Died at OUH*	74 (73%)	5 (1%)	

*See abbreviations

### TLDs

TLDs were found for 101 of the 579 patients (17%). Patients in the TLD group were significantly older and had lower Glasgow Coma Scale (GCS) scores and higher injury severity scores (AIS, ISS and NISS) than the non-TLD group. Thirty-one patients in the TLD group received an ICP monitor. For 59/101 patients, the situation was evaluated as futile, and for 42/101, it was evaluated as potentially inappropriate treatment at the time of the first decision (Table 2). The TLD group could be dichotomized into 46 cases of withholding and 55 cases of withdrawing. With regard to TLD categories, the three most frequent types were withholding neurosurgery, DNR orders and withdrawing of organ support (Table 3). Treatment limitations were set while patients were in the ICU in 80% of cases, ER in 8% and wards in 12%. In 70% of cases, the first decision regarding withholding or withdrawing life-sustaining treatment was made within 2 days after injury, while in 14% of cases, the first TLD was made later than day 7. All predefined TLD types were identified except for withdrawal of nutrition (withdrawal of nutrition after patients had been weaned off the ventilator, not including withdrawal of nutrition performed simultaneously with ventilator withdrawal). The no-escalation treatment-withholding decisions (in cases of further deterioration) were found in late cases (first TLD after day 7).

**Table 2:** Futile or potentially inappropriate treatment in the 101 patients

N=		
Futile	Physiological stabilization in the emergency room (ER) not possible	8
	Early recognition of futility (within the time frame of primary, secondary and tertiary trauma survey but after the ER)	42
	Later recognition of futility. Physiological goals could not be accomplished. Deteriorating, e.g., refractory intracranial pressure occurred after a transient initial stabilization.	9
Potentially inappropriate	Treatment started, but it might not be possible to accomplish improvement or stabilization. An effect might be accomplished but may not be wanted.	42

**Table 3:** Categories of treatment-limiting decisions in the 101 patients

Categories of treatment-limiting decisions (TLD)		N=
Withhold	Access to ICU	7
	Organ support (ventilator, vasopressor, dialysis)	10
	Neurosurgery	52
	DNR order	44
	No escalation of treatment	19
Withdraw	ICP targeted treatment	23
	Organ support	44
	Nutrition by PEG or nasogastric tube	0

* One patient may have several TLDs

Twenty percent of the first TLDs were later amended (documented reviews and changes in the plan for TLDs). Amendments involved offering neurosurgery after initial refusal for one patient and the removal of DNR orders for 3 patients, while the rest of the amendments involved a broadening of the types of TLDs when no improvement or a critical deterioration in the clinical condition occurred.

### Documentation of the decisions-making process

Table 4 gives an overview of the documentation found in the medical records with respect to key aspects of the decisions-making process when limiting or withdrawing treatment. No major conflicts between the treatment team and families regarding TLDs were registered. No involvement of the clinical ethics committees (CEC) or palliative care consults was registered.

**Table 4:** Chart documentation of key elements of the decision-making process for TLD. One patient may have several documented aspects

Documented key elements of decision-making process for TDL N=	
Prognostic statements	91
Family meetings	89
Advanced directives	0
Notifications of patient preferences regarding withholding or withdrawing of life-sustaining treatment based on communication with family	7
Notification of patient’s preferences regarding organ donation (only asked for in cases progressing towards brain death). Brain death occurred in 26 patients.	24
Multi-disciplinary discussions prior to decision	92
Documented rationale for TLD (treatment-limiting decisions)	100
Request by family to withhold/withdraw when physician recommended continued treatment	2
Request by family for continued treatment when physician recommended to WH/WD	2
Major conflict between treatment team and families regarding WH/WD	0
Involvement of clinical ethics committee (CEC)	0
Involvement of palliative care consult	0

### Mortality

**Fig. 1:**
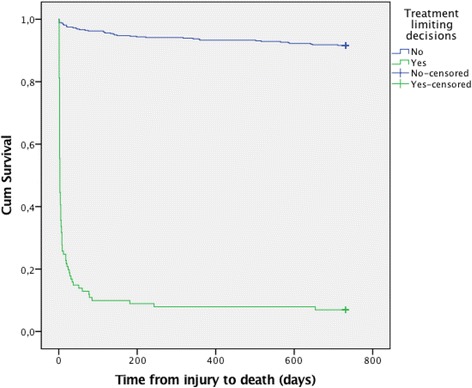
Kaplan-Meier survival plot in days for patients with TLD and without TLD

ER, in-hospital, 30-day and 2-year mortality data for the cohort are displayed in Table 5. TLDs were documented in 93% of in-hospital death cases (*n* = 79). In-hospital deaths occurred in 73% of TLD group cases and 1% of non-TLD group cases. The median time from TLD to death was 2 days (range 1–652). Time from injury to death is displayed in Fig. 1. Long-term survivors were only seen in patients grouped as “potentially inappropriate treatment” at time of first TLD. No patients with a “withdraw TLD” was discharged alive from the trauma hospital.

**Table 5:** Cohort mortality rates

	TLD-group (*N* = 101) (withhold/withdraw)	No TLD-group (*N* = 478)	All (*N* = 579)
Emergency room mortality	8 (8%) (0/8)	4 (<1%)	12 (2%)
In-hospital mortality at OUH	74 (73%) (19/55)	5 (1%)	79 (13.6%)
30-day mortality	83 (82%) (28/55)	10 (2%)	93 (16%)
2-year mortality	94 (93%) (39/55)	40 (8%)	134 (23%)

## Discussion

In this study, TLDs were found for 17% of the 579 sTBI patients. The main reasons for TLD were futility (in 58% of these cases) and potentially inappropriate treatment (in 42%). In 70% of cases, the first TLD was made within 2 days after injury. Twenty percent of the first TLDs were later adjusted, revoked in 4 patients and broadening of TLDs in 16 patients. In-hospital deaths in the TLD group and the non-TLD group occurred in 73% and 1% of cases, respectively. The median time from TLDs to death was 2 days (range 1–652). Family interaction and multi-team discussions were documented in >88% cases, but no advanced directives were found, and notifications of patients’ preferences were found in only 7% of cases.

### Prevalence of TLD

The ETHICUS study involving 31.417 intensive care patients found TLDs in 10% of cases but significant variations in practice across regions, patient factors, cultures and religions [20, 29]. TLDs were more often applied in northern than in southern European countries [20].

EoL practices in neurological and/or neurosurgical intensive care units (NICU) have been studied by other authors [30–32]. Two different single-center studies in NICU found TLDs in 4% and 14% of cases [30, 31]. A mix ICU study found TLDs in 13% of cases and noted that catastrophic brain injury was the most common reason behind TLDs [32].

A multicenter study in Canada found that the overall prevalence of TLDs for 720 sTBI patients (GCS < 9) was 22% [21]. Significant variations in mortality across centers (median 31.7%, range 10.8–44.2%) and variations in physicians’ prognostic thinking and approaches to withdrawal of life-sustaining treatment were found in connected studies [21, 22, 33].

Influential neurosurgeons have suggested regular audits of treatment limitations and mortality in sTBI [34]. Audits provide opportunities to discuss the appropriateness of TLDs in various circumstances and follow development over time. TLDs prior to death were found in 51% of cases in a 1988 audit, 68% in a 1997 audit and 89% in a 2011 audit [35].

Organ donation is somewhat interconnected with EoL practice in sTBI. A recent European union audit on a collaboration of intensive care professionals and transplant teams found large variations in EoL practice, with TLDs present in 11–73% of cases prior to death in TBI [36].

### Main reason for TLD

Guidelines advise clinicians to consider limiting treatment if continued care is not in the patient’s best interest. Decisions should be based on the available medical facts, knowledge about the natural course after injury, possible recovery trajectories, outcome data, and individualized medical assessments and guided by considerations of beneficence, non-maleficence, patient autonomy and justice [1].

No consensus has been reached about the definition of futility. The lack of a common understanding may be a source of confusion and conflict in the clinical practice setting. With the aim to avoid conflict between family and caregivers, Bosslett et al. issued a multi-society statement [10] and suggested using the concept of futility narrowly, only when physiological goals cannot be achieved. They also introduced the concept of potentially inappropriate treatment (PIT) for the more value-laden situations when there is doubt about whether goals were achievable or in the patient’s best interest [10]. There is an ongoing discussion about how potentially inappropriate interventions should be understood in TBI [37, 38]. The Society of Critical Care Medicine Ethics Committee has suggested that “*ICU interventions should generally be considered inappropriate when there is no reasonable expectation that the patient will improve sufficiently to survive outside the acute care setting, or when there is no reasonable expectation that the patient’s neurologic function will improve sufficiently to allow the patient to perceive the benefit of treatment”* [38].

### Type of TLDs

The following TLDs were registered in our study: withholding neurosurgery (52%), DNR orders (44%), withdrawing organ support (44%), withdrawing intracranial pressure-targeted treatment (23%), no escalation of treatment (19%), withholding organ support (10%) and withholding access to ICU (7%).

### Neurosurgical TLDs

Decisions about neurosurgical interventions are time-critical. If neurosurgery can improve patient outcome it is offered. Standard practice is to start monitoring intracranial pressure in all TBI cases with a GCS score <9 to be able to intervene if intracranial hypertensions develop. Only one third of patients in the treatment limitations group were ever offered pressure monitoring, a marker of initial life-saving goals. This suggests that imminent death was expected in many cases regardless of treatment effort.

### DNR

Although DNR orders were found in 44% of TLD cases, none of the patients died from a sudden cardiac arrest in the trauma hospital setting. Discussions about resuscitation in the acute care setting can be considered an indicator of a proactive strategy with regard to goal setting and as part of an effort to identify a proportionate care plan for the individual patient, as opposed to wait-and-see strategies. Discussions about DNR may help families understand the severity of the situation and prepare for the worst. It is important to be aware that sometimes, the drivers of DNR decisions may simply be doctors’ or nurses’ short-time perspectives, and there is a need to clarify when to intervene.

### Withdrawal of organ support

Postponing withdrawal of organ support for TBI patients at least 72 h after admission is recommended by the Neurocritical Care Society (NCS) to increase prognostic certainty, improve EoL care and incorporate considerations about organ donation potential [11]. Although many of the first TLDs in our study were made early (within 48 h), the mean time between the TLD and death was 2 days. This retrospective study of our current clinical practice regarding TLDs is not designed to answer whether our timing of TLDs were optimal or not. However, we acknowledge the recommendations of NCS, and will address the timing of TLDs in a later prospective study.

### No escalation of treatment

In cases with a most likely fatal TBI but still with a small possibility of survival, we found that the no-escalation treatment decision was made. This decision allows following conditions over time and simultaneously setting a limit, as opposed to an open-ended strategy. This decisional type of limitation is still debated [17, 18, 39].

### Withholding access to ICU

Withholding access to the ICU was in our study not related to advanced directives. Transfers of patients directly from the ER to the ward for palliative care seldom occurred. A concern about unwanted aggressiveness in treatment approaches for some patients might be raised, as 25% of all admitted patients were > 69 years old. The high number of ICU admittances (85%) must be interpreted as an approach to giving a treatment trial to all admitted patients regardless of age.

### Adjusted TLDs

Initial clinical and radiological assessment is often performed when analgesia and sedation are still confounding the neurological assessment. Early decisions may be wrong and need to be revised or reversed later [13]. Some decision types are reversible (no access to ICU, no surgery, DNR orders, no escalation of treatment), and others are irreversible (withdrawing organ support). Time is needed for repeated evaluations before the initial clinical impression should be acted on. We found adjusted TDLs regarding DNR orders, surgery and goals of care. In our study the first TLD in 20 patients were later adjusted, revoked in 4 patients and broadening of TLDs in 16 patients.

### Timing of TLDs

Early decisions dominated our data. Only 14% of TLDs were made after the first week.

The appropriate timing for discussions about TLD in TBI should be assessed on individual bases, dependent on patient and family factors. Doctors may feel reluctant to discuss non-treatment options with families for fear of anger and distress [40].

### Adherence to guidelines in decision-making processes

In a multicenter study in Canada, patients’ wishes, as indicated by the family, were found to influence TLDs in 34% of cases [21]. We found patients’ preferences (regarding continuing, limiting or withdrawing care) in only 7% of cases and no advanced directives. According to Norwegian legislation and National ethical guidelines physicians are obliged to explore patient’s preferences through dialog with patient’s family prior to decision-making for patients lacking capacity, to evaluate patient’s best interest and to document medical and value considerations behind decisions. The lack of documentation of value considerations concerns us and will be followed up. In Canada, the legal requirement for consent from families for patients without a decision-making capacity differs from the Norwegian legislation and may explain differences in chart documentation practices [41].

Notably, a randomized controlled study showed that patients’ values had little impact on physicians but that the provision of prognostic estimates increased their willingness to discuss the level of care [42]. We found prognostic statements in nearly all cases prior to treatment limitations, but they often used vague language.

We did not find any conflict or involvement of palliative care consults or clinical ethics committees. A study exploring whether TBI cases were clear, difficult or very difficult found that only 1% of cases were perceived to be very difficult and involve ethical consults [43]. A single-center study of a NICU in the US showed that most surrogate decision-makers were comfortable with the process of withdrawing care [31].

## Limitations

Retrospective design has clear limitations with regard to methods of studying ethical questions in clinical practice.

## Conclusions

In this study, TLDs were found in 17% of sTBI patients. The main reasons for TLD were futile treatment (in 58% of these cases) and potentially inappropriate treatment (in 42%). In 70% of cases, the first TLD was made within 2 days after injury. The most frequent categories of TLDs were withholding neurosurgery, DNR orders and withdrawal of organ support. In-hospital deaths in the TLD group and the non-TLD group occurred in 73% and 1% of cases, respectively. Improvements in clinicians’ documentations of TDLs are needed. Value considerations in TLDs need to be further explored.

## Abbreviations

AIS: Abbreviated Injury Scale
CEC: Clinical ethics committee
DNR: Do not resuscitate
EoL: End-of-life
ER: Emergency room
EVD: External ventricular drainage
GCS: Glasgow Coma Scale
ICU: Intensive care unitICP Intracranial pressure
ISS: Injury Severity Score
LOS: Length of stay
NISS: New Injury Severity Score
OUH: Oslo University Hospital
PIT: Potentially inappropriate treatment
TBI: Traumatic brain injury
TLD: Treatment-limiting decision
TR-OUH: Oslo University Hospital Trauma Registry
